# Deep learning-enabled high-speed, multi-parameter diffuse optical tomography

**DOI:** 10.1117/1.JBO.29.7.076004

**Published:** 2024-07-19

**Authors:** Robin Dale, Biao Zheng, Felipe Orihuela-Espina, Nicholas Ross, Thomas D. O’Sullivan, Scott Howard, Hamid Dehghani

**Affiliations:** a University of Birmingham, School of Computer Science, Medical Imaging Lab, Birmingham, United Kingdom; b University of Notre Dame, Department of Electrical Engineering, Notre Dame, Indiana, United States

**Keywords:** deep learning, diffuse optical tomography, frequency domain, scattering, breast imaging

## Abstract

**Significance:**

Frequency-domain diffuse optical tomography (FD-DOT) could enhance clinical breast tumor characterization. However, conventional diffuse optical tomography (DOT) image reconstruction algorithms require case-by-case expert tuning and are too computationally intensive to provide feedback during a scan. Deep learning (DL) algorithms front-load computational and tuning costs, enabling high-speed, high-fidelity FD-DOT.

**Aim:**

We aim to demonstrate a simultaneous reconstruction of three-dimensional absorption and reduced scattering coefficients using DL-FD-DOT, with a view toward real-time imaging with a handheld probe.

**Approach:**

A DL model was trained to solve the DOT inverse problem using a realistically simulated FD-DOT dataset emulating a handheld probe for human breast imaging and tested using both synthetic and experimental data.

**Results:**

Over a test set of 300 simulated tissue phantoms for absorption and scattering reconstructions, the DL-DOT model reduced the root mean square error by 12%±40% and 23%±40%, increased the spatial similarity by 17%±17% and 9%±15%, increased the anomaly contrast accuracy by 9%±9% ($\mu_{a}$), and reduced the crosstalk by 5%±18% and 7%±11%, respectively, compared with model-based tomography. The average reconstruction time was reduced from 3.8 min to 0.02 s for a single reconstruction. The model was successfully verified using two tumor-emulating optical phantoms.

**Conclusions:**

There is clinical potential for real-time functional imaging of human breast tissue using DL and FD-DOT.

## 1 Introduction

### 1.1 Diffuse Optical Tomography

Diffuse optical tomography (DOT) is a safe and non-invasive medical imaging technique in which near-infrared (NIR) light (600 to 1000 nm) is transmitted between an array of sources and detectors positioned on the surface of the body and the properties of the measured light are used to reconstruct images of the optical properties of the intervening tissue. DOT has been successfully applied in numerous research areas requiring characterization of tissue composition and metabolism, including neuroimaging^1^^,^^2^ and musculoskeletal^3^ imaging, and has been proposed as an alternative or complementary technique to X-ray mammography for clinical breast imaging.^4^ Specifically, as the spectral attenuation of NIR light is strongly dependent on tissue physiology, DOT-measured biomarkers can be used to detect tumors,^5^ distinguish malignant tumors from benign tumors,^6^^,^^7^ and predict individual responses to neoadjuvant chemotherapy,^8^ without exposing the patient to ionizing radiation.

In DOT, the optical properties of tissue are described via two spectrally varying parameters: absorption ($\mu_{a}$) and reduced scattering (${\mu_{s}}^{\prime }$). It is generally agreed that utilizing continuous-wave (CW) data (intensity only) leads to a non-unique problem whereby only absorption-related images can be derived, by assuming prior knowledge about tissue scattering. The incorporation of time-of-flight information via either phase measurement—as in frequency domain (FD)—or a temporal point spread function—as in time domain (TD)—allows for an estimation of the photon propagation path, thereby enabling recovery of absolute values for both $\mu_{a}$ and ${\mu_{s}}^{\prime }$. When used for functional neuroimaging [as in near-infrared spectroscopy (fNIRS)], FD measurements can provide a more accurate assessment of functional changes compared with CW^9^ as well as an improved spatial resolution in DOT reconstructions.^10^ The ${\mu_{s}}^{\prime }$ parameter is additionally indicative of tissue microstructure and can provide an additional biomarker for distinguishing malignant versus benign breast tumors.^11^^,^^12^ Clinical adoption of frequency-domain diffuse optical tomography (FD-DOT) has so far been limited due to the size and complexity of the systems; however, recent hardware developments have significantly improved miniaturization, accuracy, and usability,^9^ enabling accurate, high-speed, and depth-sensitive broadband FD-NIRS measurements at multiple modulation frequencies with a hand-held probe^13^ and thereby high-speed two-dimensional (2D) imaging of bulk optical properties.^14^

Reconstructing three dimensional (3D) as opposed to 2D images using DOT provides important additional information regarding the size, shape, and position of features such as tumors. This approach is ill-posed as the number of reconstruction voxels is generally far greater than the number of available measurements. 3D DOT has primarily been performed using computationally intensive model-based optimization algorithms,^15^ whereby a “forward model” of the light propagation in the tissue being imaged is used to estimate the internal, spatially varying optical properties by matching the modeled data to the measured data. For specific applications in breast imaging, a range of different and complementary approaches have been developed to overcome the ill-posedness and under-determined nature of this optimization approach; these include the use of the generalized least square,^16^ structural priori,^17^ and spectral constraint methods.^18^ A detailed review of utilizing prior knowledge within image recovery and analysis of DOT data for breast imaging has highlighted that differing types of constraints, each having its advantages as well as drawbacks, can be used.^19^ Nonetheless, it is generally accepted that image reconstruction in DOT is a challenging problem due to the nonlinear nature of light propagation in tissue that requires user-adjusted regularization parameters, which can be case-specific and difficult to generalize.

### 1.2 Deep Learning Diffuse Optical Tomography

DOT can also be performed using multi-layered neural networks, trained using deep learning (DL). In the past decade, DL has revolutionized multiple industries and research areas—notably computer vision and natural language processing—with the ability to approximate and learn arbitrarily complex functions without the need for explicit analytical modeling.^20^ Given sufficient training data, DL models tend to outperform computational algorithms that rely on manually designed features or heuristic data processing techniques because they autonomously capture complex relationships in the data that are statistically relevant to the problem and often difficult for engineers to conceptualize and design. In DL-DOT, the inverse imaging problem is solved directly by learning a statistical mapping between the measurements and optical properties from a large labeled dataset.^21^ Because the availability of ground-truth labels of human tissue is prohibitively limited, photon simulation software packages such as NIRFAST^15^ or Monte Carlo^22^ can be used along with a realistic noise and calibration model to generate examples to train and test the DOT models.

DL-DOT has been repeatedly demonstrated with 2D geometries using simply designed neural networks.^23^^,^^24^ However, reconstructing in 2D places a fundamental constraint in a clinical context as the size and shape of anomalies such as tumors cannot be accurately characterized. Only a few published studies have demonstrated 3D DOT using DL. Zou et al.^25^ proposed a fully connected (FC) encoder–decoder architecture with structural constraints derived from a physical model (born constraint) and a co-registered ultrasound image to solve the forward and inverse DOT problems based on a high-density reflectance probe for breast imaging. The hybrid model was found to outperform both standard born-conjugate gradient descent and the unconstrained version of the network. Yoo et al.^26^ used the convolutional framelet theory to demonstrate how a model comprised of an FC layer followed by a 3D convolutional neural network (CNN) with an encoder–decoder structure can theoretically invert the Lippmann–Schwinger integral equation,^26^ thereby providing justification for such an architecture for modeling photon propagation and solving the DOT inverse problem. Deng et al.^27^ found that extending this architecture by adding a separately trained U-Net improved anomaly localization and contrast. The model they presented—“FDU-net” (so named for the Fully-connected, encoder-Decoder, and U-Net modules)—was the first DL-DOT model trained exclusively on simulated data and demonstrated *in vivo* for 3D imaging of a human breast, thereby demonstrating the viability of this approach for the clinical application of DL-DOT. Notably, the FDU-net architecture is generalizable to any probe design, imaging geometry, and measurement type, and although other architectures have been proposed,^28^ they have not yet been theoretically grounded or demonstrated with *in vivo* data. Direct comparisons of DL-derived DOT images to model-based DOT have consistently shown improved reconstruction quality, including reduced artifacts and greater optical contrast, which are crucial for effective clinical use.^26^^,^^27^^,^^29^

The other key benefit of DL-DOT is reconstruction speed; although the geometry-specific data generation and training process for DL-DOT are computationally expensive, once the model is trained, inference can be orders of magnitude faster than with iterative finite element model (FEM)-DOT because the feed-forward function of a neural network consists primarily of matrix multiplications and additions, which are extremely fast to compute.

So far, the majority of published DL-DOT models (including all of those mentioned above) have used CW measurements to predict either relative changes or quantitative values of $\mu_{a}$. Imaging relative $\mu_{a}$ enables functional analysis of tissue composition but is clinically limited as it cannot facilitate longitudinal or inter-subject comparison. Quantitative $\mu_{a}$ reconstruction using CW only faces a physical limitation to its accuracy due to the difficulty of separating the attenuating effects of absorption from those of scattering.^30^ It is a common practice to assume constant scattering—known to be unrealistic in human tissue imaging—resulting in unreliable measurements.^31^ Often, the same assumption has been incorporated in the design of simulated DL-DOT datasets by setting a constant known homogeneous scattering value across all training examples, which inevitably enhances the accuracy of the trained model for volumes in which the ${\mu_{s}}^{\prime }$ value matches the training value and introduces errors in cases in which it does not. Even in cases in which the scattering values of tissue models used to generate training data have been randomized according to a biologically relevant range, DOT-recovered absolute $\mu_{a}$ is likely to be unreliable when utilizing CW data without a direct indicator of scattering.

A few studies have investigated incorporating time-of-flight information to enhance DL-DOT. Takamizu et al.^32^ presented a novel approach for DL-DOT with TD measurements, in which a “long short-term memory” model trained on the temporal point spread function was used to predict the position of an absorbing anomaly in a 2D tissue model with high accuracy. Because they transformed the DOT inversion into a classification problem, by assuming an anomaly with a known size and optical properties and a known range of discrete possible positions, this method cannot be directly compared with the published 3D DOT results using CW data. Murad et al.^33^ demonstrated end-to-end DL-DOT with FD data to reconstruct 2D images of $\mu_{a}$ and ${\mu_{s}}^{\prime }$ simultaneously, hypothetically enabling assessment of both functional and structural indications of tissue health with a single scan. They trained one-dimensional and 2D CNN architectures using simulated measurements based on a radial transmission geometry and successfully tested the model with a series of circular, breast tissue-simulating phantoms. Based on the reconstructions presented, their DL models achieved significant improvement in accuracy in terms of recovered contrast and anomaly size for both $\mu_{a}$ and ${\mu_{s}}^{\prime }$ compared with model-based reconstruction; however, some of the reconstructions indicate a significant degree of crosstalk (CT) between $\mu_{a}$ and ${\mu_{s}}^{\prime }$ as the recovered value of one parameter is significantly higher than the true anomaly value while the other is significantly lower.

In this work, a novel DL-DOT model, based on the FDU-net architecture, is designed and demonstrated to simultaneously reconstruct 3D absolute values of both $\mu_{a}$ and ${\mu_{s}}^{\prime }$ directly from FD-NIR measurements. A simulated FD-DOT dataset based on a single-source/multi-detector probe configuration and linescan protocol is used for training to exploit the specific benefits of combining the high-speed DL-DOT with the relative flexibility and comfort afforded by modern handheld FD-NIRS systems.

The proposed model is evaluated via clinically relevant metrics in comparison to a model-based reconstruction algorithm. A detailed description of the dataset, model design and training procedures is provided in Secs. 2.1–2.4, followed by an analysis of the reconstruction results in Sec. 3, is provided to assess the pros and cons of DL-FD-DOT and the feasibility of utilizing the technique as a point-of-care tool for breast imaging.

## 2 Methods

### 2.1 Simulated FD-DOT Data

**Fig. 1:**
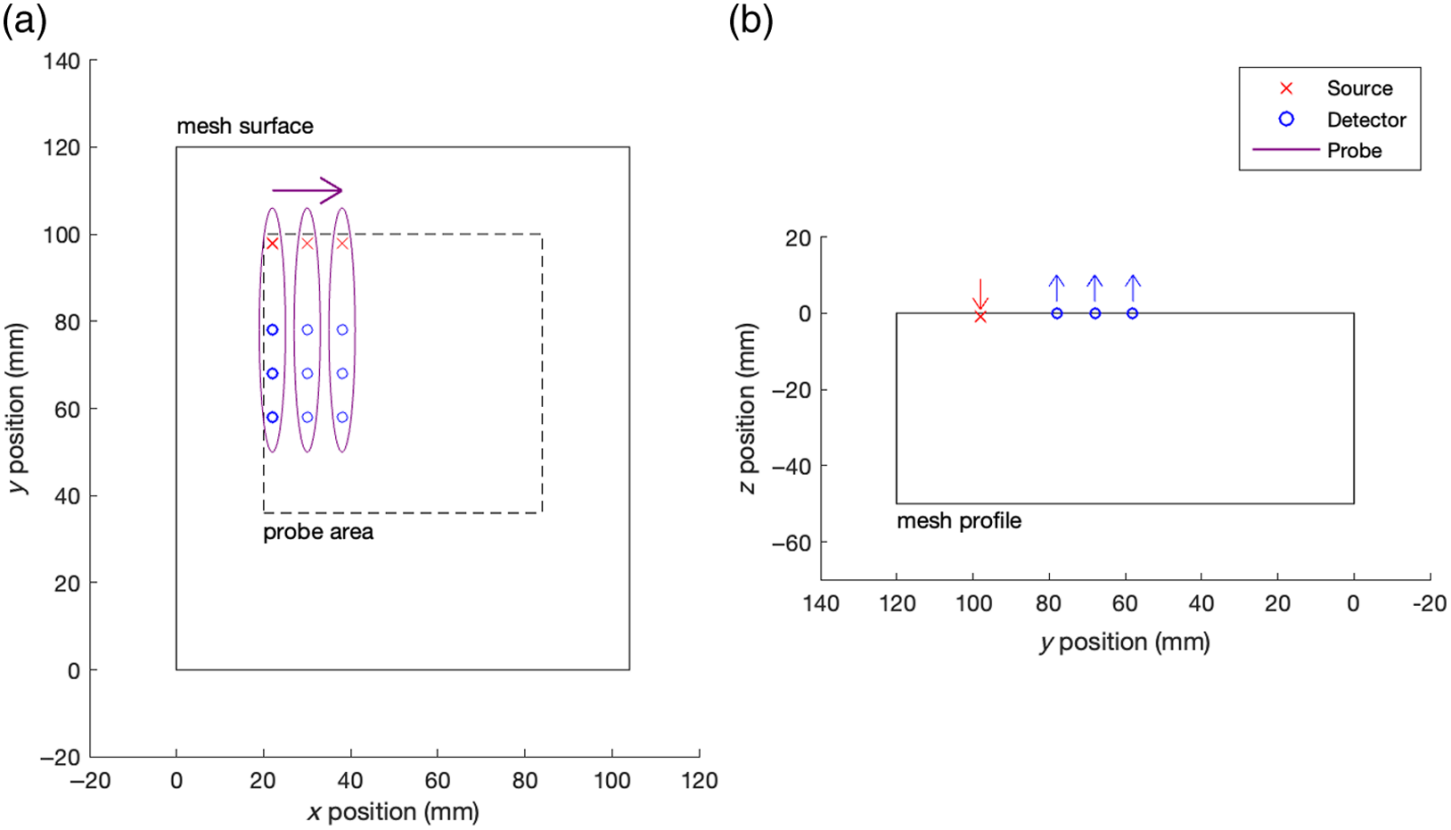
Schematic of model and scanning protocol. (a) Top view, (b) side view.

To train and evaluate the DL model, synthetic FD-DOT data were simulated using the NIRFAST Matlab package. A [104×120×50  mm] cuboid finite element model (FEM), consisting of 104,039 nodes comprising 598,181 tetrahedral linear elements, was generated. A total of 576 source/detector pairs were positioned on the mesh surface to emulate a probe with a single source, and detectors were positioned at 20, 30, and 40 mm from the source, scanning horizontally across the top surface of the model, with measurements taken at 4 mm increments, for 5 s per position, over a defined scan region of 64×64  mm (Fig. 1).

Amplitude and phase boundary measurements were computed for each source/detector pair using NIRFAST based on the diffusion approximation,^15^ yielding a vector of 1152 boundary measurements per simulation. For each training example, a random set of 0 to 5 anomalies was added to the model; each was characterized by a local perturbation in $\mu_{a}$, ${\mu_{s}}^{\prime }$, or both. A system-derived, amplitude- and modulation frequency-dependent noise model^34^ was used to add realistic noise to each example. A total of 11,000 noise-added examples were generated and then divided into 10,000 training and 1000 validation examples. To emulate real-world variability, the optical properties of each training volume were randomized within biologically realistic distributions based on population studies.^12^^,^^35^^,^^36^ The number, shape, size, and position of anomalies were randomized within the values reported in Table 1.

**Table 1:** Parameters of the tissue-emulating DOT dataset.

Background $\mu_{a}$	$0.005+-0.002\;{\mathrm{mm}}^{-1}$
Background ${\mu_{s}}^{\prime }$	$0.98+-0.20\;{\mathrm{mm}}^{-1}$
$\mu_{a}$ anomaly contrast	1.5 to 3.5
${\mu_{s}}^{\prime }$ anomaly contrast	1.5 to 2.5
Number of anomalies	0 to 5
Anomaly radius	5 to 15 mm
Anomaly shape	Sphere, disk, cuboid
Anomaly depth	0 to 20 mm

No data augmentation techniques were considered in this work as absolute tomographic imaging is nonlinear in nature, and therefore, data augmentation becomes more challenging, whereas in linearized models, such as fluorescence molecular tomography,^37^ this may be more appropriate to consider.

The simulated data are publicly available at https://doi.org/10.5281/zenodo.10379351.

### 2.2 Experimental FD-DOT Data

Two physical phantoms with different optical properties were used to test the DOT models. The details of the estimated optical properties of the phantoms are shown in Table 2.

**Table 2:** Details of the two optical phantoms measured.

	Phantom 1	Phantom 2
**Background OPs** (${\mathrm{mm}}^{-1}$)	830 nm: $\mu_{a}=0.0103$, ${\mu_{s}}^{\prime }=1$	830 nm: $\mu_{a}=0.0024$, ${\mu_{s}}^{\prime }=1.045$
		690 nm: $\mu_{a}=0.0044$, ${\mu_{s}}^{\prime }=1.369$
**Anomaly OPs** (${\mathrm{mm}}^{-1}$)	830 nm: $\mu_{a}=0.1$, ${\mu_{s}}^{\prime }=1$	830 nm: $\mu_{a}=0.0049$, ${\mu_{s}}^{\prime }=1.849$
		690 nm: $\mu_{a}=0.01$, ${\mu_{s}}^{\prime }=2.35$
**Anomaly shape**	Horizontal cylinder	Vertical cylinder
**Anomaly size** (mm)	Radius = 5, length = 15	Radius = 10, height = 45
**Anomaly in depth** (mm)	12.5	5

The first phantom (phantom 1) has been described and used in previous work^38^ and has a spatially adjustable, highly absorbing anomaly, which was positioned in the center of the phantom for the entirety of this study.

The second phantom (phantom 2) was designed and fabricated for this study to have more biologically relevant optical properties for the application of breast imaging. Phantom 2 was comprised of P4 silicone rubber (Eager Polymers, Chicago, Illinois, United States) and included water-soluble nigrosin dye and anatase titanium (IV) oxide (${\mathrm{TiO}}_{2}$) (MilliporeSigma, St. Louis, Missouri, United States) to mimic absorption and scattering features, respectively. To begin, 276.6  μL of nigrosin solution (10  mg/1  mL $H_{2}O$) was added to 60 g of P4 activator in a glass beaker. The contents were then mixed with ultrasonication [Branson Ultrasonics SFX550, Danbury, Connecticut, United States)] for 5 min. After sonication, the components were further mixed with a magnetic stir bar at 300 RPM for 3 min. This process was repeated three times to ensure that the nigrosin was fully incorporated into the activator. Following this step, 1.6187 g of ${\mathrm{TiO}}_{2}$ was combined with the nigrosin mixture, and the sonication procedure was repeated. In between each sonication, a metal stirring rod was used to mix the solution and scrape any coagulated ${\mathrm{TiO}}_{2}$ from the walls of the beaker. The sonication process was repeated three times to create a homogeneous solution. Once complete, the P4 base and activator mixture were combined in a separate container. Using an electric hand mixer (VonHaus VonShef five-speed electric mixer, Salford, United Kingdom) at the highest speed, the container’s contents were stirred for 3 min. The container was then placed into a $0.31\;{\mathrm{ft}}^{3}$ vacuum chamber and degassed for ∼20  min. Finally, a portion of the mixture was poured into a 3D-printed cylindrical mold to make the inclusion. The remaining container contents were formed into a homogeneous reference phantom. After curing for 24 h, the inclusion was removed from the mold, placed upright into the center of a new plastic container, and secured to the bottom using a thumbtack that was inserted on the bottom’s outside and covered in epoxy to ensure no leakage. To make the surrounding background medium, the aforementioned procedure was repeated using 116.2  μL of nigrosin solution and 0.9002 g of ${\mathrm{TiO}}_{2}$. In the final step, the mixture was degassed for 10 min to remove a majority of the air bubbles, transferred into the container with the inclusion, and degassed for an additional 10 min. The container was removed from the vacuum chamber and moved to a flat, level surface to cure for 24 h.

#### 2.2.1 Experimental data collection

**Fig. 2:**
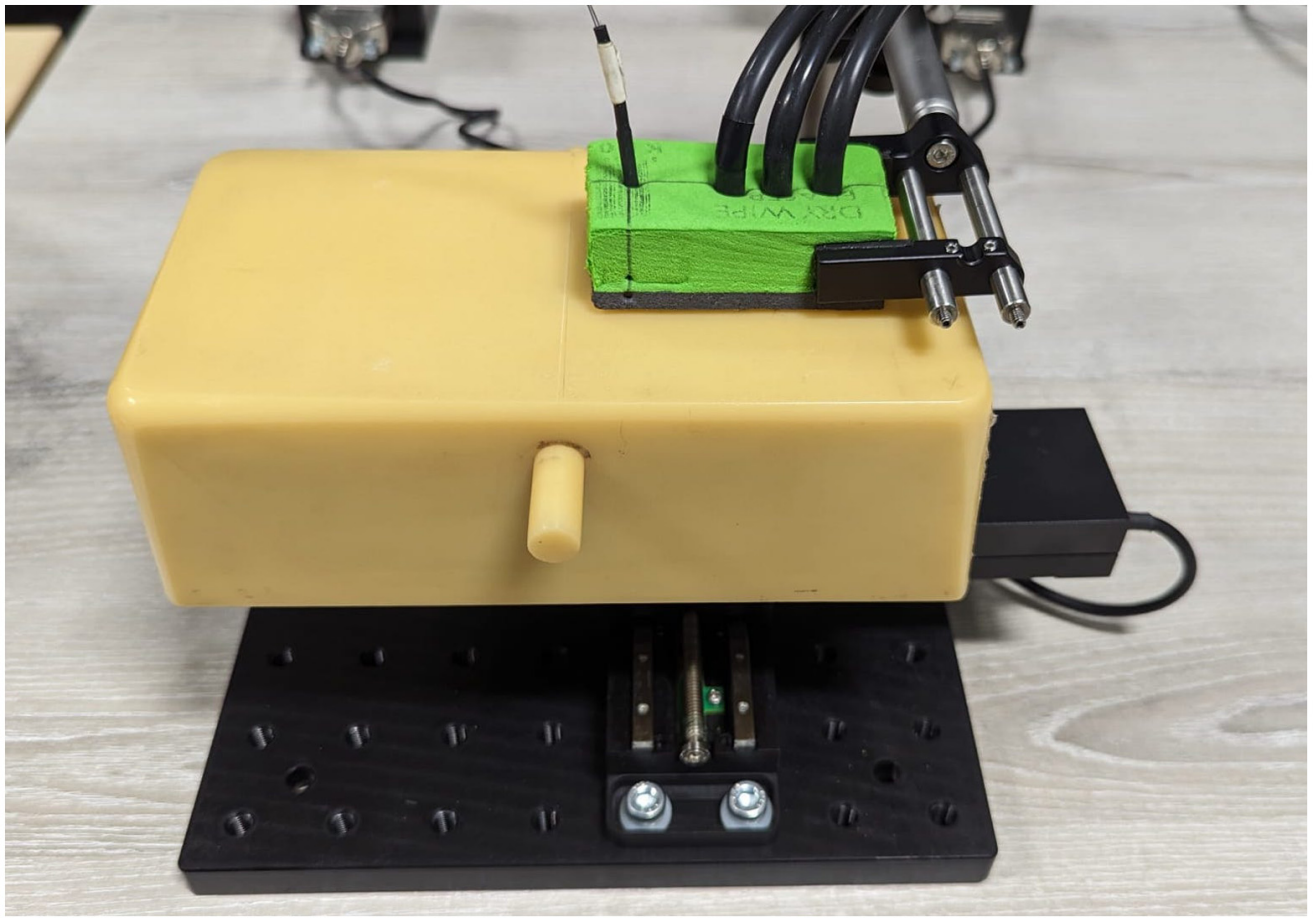
Phantom 1 attached to an automated probe holder.

**Fig. 3:**
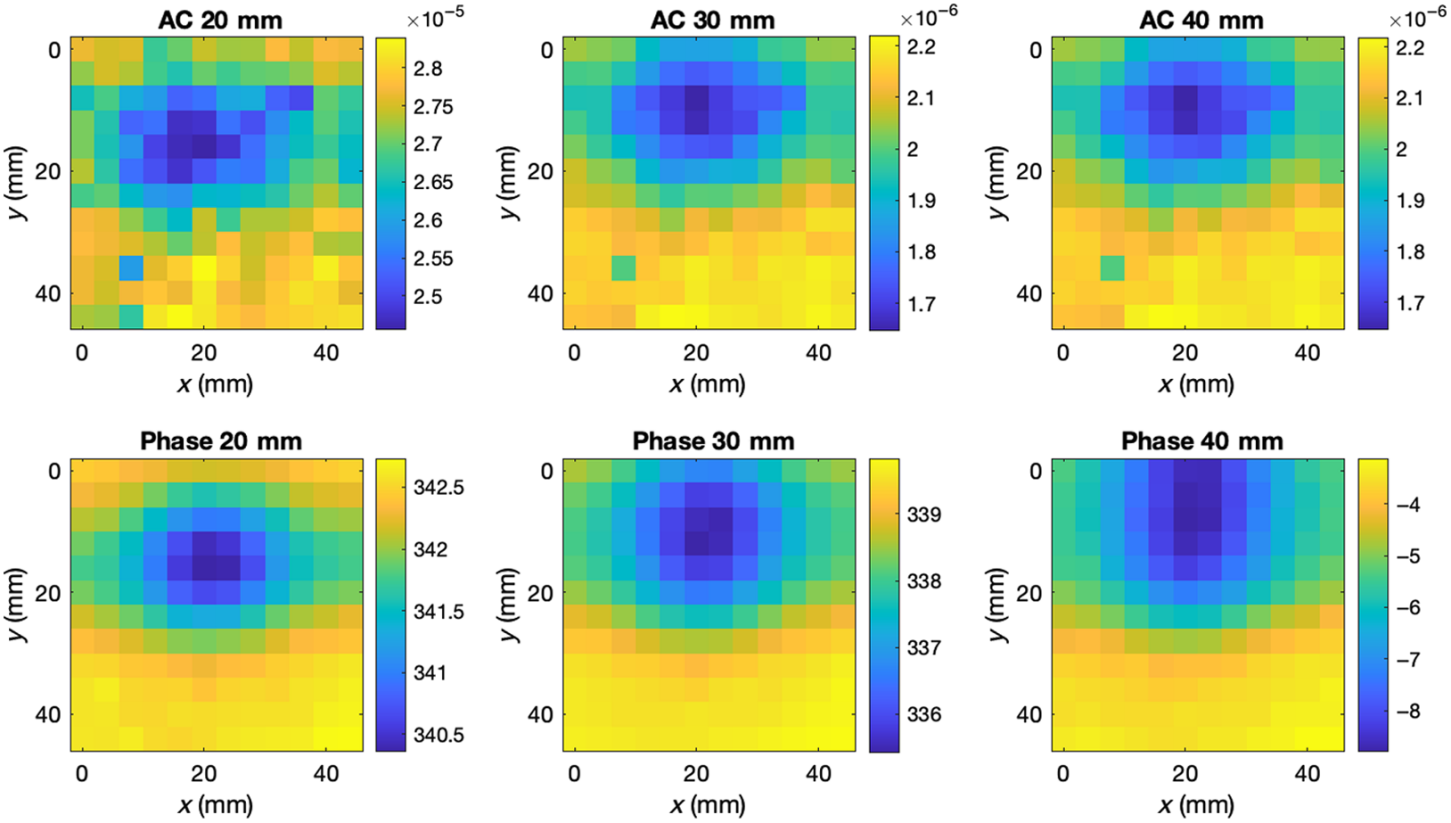
Topograhic maps of amplitude (AC) and phase data at three source/detector separations, collected from the optical phantom shown in Fig. 2.

FD-DOT measurements were collected using an ISS Imagent V2 system. Two, 140 MHz-modulated, laser diodes (sources, 830 and 690 nm) and three photo-multiplier tubes (detectors) were positioned linearly within a secure foam holder, at source/detector separations of 20, 30, and 40 mm. Each phantom was attached to a programmable x-y-stage beneath the probe holder (Fig. 2). AC amplitude and phase data were collected while the phantom was moved over a 48×48  mm scan region at 4 mm increments, generating a 12×12  mm measurement grid. The 830 nm amplitude and phase data from phantom 1 are shown in Fig. 3.

### 2.3 Model-Based DOT Algorithm

To explore and demonstrate the differences and trade-offs between DL and model-based reconstruction algorithms for multi-parameter recovery, NIRFAST, a FEM-based optimization algorithm, was utilized; it is based on the method described fully in Ref. 15 and is henceforth referred to as “FEM-DOT.” Briefly, photon propagation is modeled using the diffusion approximation to predict boundary measurements based on the initial estimation of optical properties, which is then iteratively updated by

$$J^{T}(JJ^{T}+\lambda I{)}^{-1}J^{T}\delta \Phi =\delta \mu ,$$ (1)

where δμ is the voxel-wise update vector of the optical properties for the current iteration; δΦ is the projection error (difference between measured data and current iteration); λ is the regularization factor; and J is the Jacobian sensitivity matrix, $J\in R_{\left[n\times m\right]}$, where n is the number of boundary measurements (intensity and phase for each source/detector pair) and m is the number of reconstruction unknowns ($\mu_{a}$ and ${\mu_{s}}^{\prime }$ for each voxel). J defines the first-order derivative of the measurements with respect to the optical property values, that is, the rate of change of data due to a small change in optical properties, which is calculated using the adjoint method.^39^ To avoid committing “inverse crime,”^40^ a secondary phantom mesh was generated for iterative reconstruction; it has the same dimensions and source/detector positions as the data generation mesh described in Sec. 2.1, but with slightly higher density and differing node placement.

The initial value of the regularization term λ was set to 0.01 of the maximum value of the diagonal of $JJ^{T}$ and was reduced by a decay factor of $10^{0.25}$ at each iteration, and the process was continued until the projection error between two subsequent iterations did not improve below the convergence threshold, set at 2%. It is worth noting that the regularization parameter *lambda*, while used to allow for the inversion of $JJ^{T}$, also acts as a smoothing function: a lower value produces a higher-resolution parameter recovery, which can also suffer from over-fitting, whereas a higher value produces a much lower-resolution image due to under-fitting. The initial λ value was chosen from a logarithmic range of possible values (0.0001, 0.001, 0.01, 0.1, 1) to minimize the root mean square error (RMSE) over a subset of the validation examples, with the decay factor selected based on prior experience. Although it is possible to optimize regularization for each test example by selecting a value to minimize the residuals of either the predicted optical properties or the modeled measurements, this was not done in this work as it would require prior knowledge that may not be available in the context of clinical DOT (in the optical property case) or extensive evaluation at each iteration for each reconstruction (in the measurement case) using, for example, the L-curve method. For this reason, a single λ value was used for all test cases in this study.

A reconstruction basis of 4 mm voxels was used; this was also the basis used for the DL-based DOT, and at each iteration, an 8×8×8  mm Gaussian filter was applied to each of the $\mu_{a}$ and ${\mu_{s}}^{\prime }$ updates, chosen ad hoc based on a visual inspection of a reconstructed validation image.

Prior to inversion, the Jacobian was row- and column-normalized, to account for the dynamic range of data, as well as the optical properties.

### 2.4 DL-DOT Algorithm

#### 2.4.1 Data preprocessing

The noise-added intensity measurements were converted to log amplitude ($\log_{e}\;\mathrm{AC}$) and then, along with the phase measurements in degrees, were “min-max” normalized by subtracting the minimum and dividing by the range of each measurement across all training examples. This step ensures that each input to the network has a minimum of 0 and maximum of 1 across the entire training set, leading to equally scaled gradients and therefore more stable convergence during training. This is especially important with FD data because the two types of measurement—amplitude and phase—differ significantly in terms of magnitude and distribution of the measurements. The original FDU-net paper^27^ used min-max normalization to preprocess the intensity data; however, in this work, models trained with z-score input data were found to perform better (faster convergence and reduced error in reconstructions) compared with those trained with min-max normalization. This is thought to be due to extreme outliers in the minimum and maximum values of some phase measurements—caused by system noise—which distort the distribution when used as a normalization factor. Z-score was therefore used in place of min-max for the final model evaluated in Sec. 3. The target $\mu_{a}$ and ${\mu_{s}}^{\prime }$ volumes were also independently min-max normalized, using the global minimum and maximum values for each parameter across the training set. The minimum and range of values used in preprocessing were saved and used to apply the same preprocessing transformation to the unseen test data and to reverse the transformation on the model’s predictions.

To reduce the training time and create comparable output to the FEM-DOT algorithm, the target volumes were down-sampled to 4 mm resolution using bicubic interpolation. In addition, voxels outside the scan region (Fig. 1) and those greater than 32 mm in depth from the surface were removed, resulting in a target volume with shape [16×16×8×2] (H×W×D×n_channel) for each example, where n_channels refers to the two optical parameters, $\mu_{a}$ and ${\mu_{s}}^{\prime }$. Due to the physical limitations, neither DOT algorithm is expected to reconstruct accurately below 32 mm deep (given a maximum source/detector separation of 40 mm) or in areas without sufficient sensitivity; however, it is important to include these regions in the simulation stage to avoid edge effects on the photon fluence.

#### 2.4.2 Neural network architecture

The CNN architecture used in the DL algorithm was adapted from the FDU-net model published in Ref. 27. The model has three distinct modules: an FC layer, a convolutional encoder–decoder network, and a U-Net. The FC layer performs an initial mapping from measurement space to vector space and is given by

Y=WX+B, (2)

where X is the input vector; W and B are the weight matrix and bias vector, respectively; and Y is the layer output. This layer performs a function somewhat analogous to the inverted Jacobian sensitivity matrix used in FEM-DOT to calculate the prediction update at each iteration as it encodes the relative sensitivity of each measurement value to each optical property at each voxel. However, although the Jacobian is specific to a given set of optical parameters—calculated using the diffusion approximation—the FC weights and biases constitute a generalized inverse operator learned from the entire training data.

The CNN encoder–decoder module applies a series of 3D convolutions to the initial inverse estimate; in each, a set of four-dimensional filters (three spatial dimensions plus a channel dimension that represents $\mu_{a}$ and ${\mu_{s}}^{\prime }$ in the first layer and extracted features in all subsequent layers) is cross-correlated with the input over the three spatial dimensions. The U-Net module performs additional 3D convolutions with max-pooling layers in between, followed by symmetric up-sampling performed by transposed convolutions. At each stage of upsampling, the feature maps with the same dimensions from the downsampling stage are concatenated with the output along the channel dimension (known as “skip” connections), allowing for integration of high-level and low-level features. In both the CNN and U-Net modules, the stacked convolutional layers enable hierarchical feature detection and denoising, where the features and noise characteristics are learned automatically from the training data. By contrast, the spatial filtering performed in the conventional FEM method relies on manually designed filters (a Gaussian filter as used in this work) and matrix regularization (λ) for denoising.

In the adapted model used here for multi-parameter reconstruction, the input contained both log amplitude and phase measurements for each channel (source/detector pair), combined in a single vector. The two target volumes, corresponding to the two optical parameters, $\mu_{a}$ and ${\mu_{s}}^{\prime }$, were treated as two channels of a single volume—similarly to the RGB channels in an image—rather than predicted separately, to preserve spatial coherence between parameters and allow the model to extract complex relational features. Therefore, in the first and last layers of the network, the channel dimension represents the two optical parameters $\mu_{a}$ and ${\mu_{s}}^{\prime }$, whereas in each of the intermediate layers, it represents the number of feature maps, determined by the number of filters used in the previous convolutional layer.

#### 2.4.3 Region of interest (ROI) targeted loss function

As the simulated tissue volumes are dominated by background voxels, with anomalies making up an average of just 11.3%±7% of voxels across the training examples, using a conventional reconstruction error function, e.g., RMSE, which weights all voxels equally, can lead to slow convergence and poor reconstruction contrast, especially in smaller anomalies. To avoid this issue, a region-of-interest weighted mean-squared-error (${\mathrm{MSE}}_{\mathrm{ROI}}$) loss function, also adapted from Ref. 27, was used to train the models; it is given by

MSEROI=WROI·‖y^ROI−yROI‖+(1−W)·‖y^bg−ybg‖, (3)

where $W_{\mathrm{ROI}}$ is the ROI weight, y is the ground truth label, and y^ is the model prediction. The ROI weight parameter determines the weight of the loss function at voxels within tumor-emulating anomalies (indicated by an ROI mask) relative to the background voxels. Because the updates to the model’s weights and biases during training are calculated using the back-propagated differentials with respect to the loss function, setting this value between 0.5 and 1 leads to relatively larger updates for voxels contributing to anomaly-related features, leading to faster convergence and greater contrast in the recovered values. Furthermore, because the 3D convolutional layers impose positional invariance in the feature extraction, the ROI loss function improves the detection of anomaly-related features regardless of their position in the volume. This technique is a form of “privileged knowledge,” meaning that information that is available in the training stage but not the testing stage—the precise position of anomalies in this case—can be used to improve the performance of the model. In the FEM-DOT algorithm, prior knowledge of the spatial distribution of optical properties can also be used to improve reconstruction, but it has to be explicitly incorporated, either in the initial reconstruction estimate or via customized spatial constraints, both of which can be difficult to formulate, often rely on an expert’s intuition, and can prevent accurate convergence if they are incorrect.

Because the dataset in this study included anomalies in which either absorption or scattering was perturbed, as well as anomalies in which both were perturbed, the ROI masks were different for each parameter and were represented as two-channel volumes with the same dimensions as the target and reconstruction volumes. Here, it is proposed that $\mu_{a}$ and ${\mu_{s}}^{\prime }$ ROI masks for each example be combined by a voxel-wise logical or operation and the single resulting mask be applied to the loss for both channels of the network output, thereby penalizing CT error between the two parameters in anomaly regions. In a direct comparison between otherwise identically trained models, this approach was found to reduce the mean squared error (MSE) by an average of 25% in examples with single-parameter perturbations. The ROI weight parameter was not included in the hyper-parameter grid search, and the best value was determined through trial and error to be approximately the ratio of background voxels to total voxels across the entire training dataset ($W_{\mathrm{ROI}}=0.887$).

#### 2.4.4 Hyper-parameter search

The optimal hyper-parameters for a DL model, balancing fitting power with regularization and generalizability, are generally difficult to predict a priori because they depend on the nature of the function being approximated, the number and distribution of available training examples, and the amount of noise in the data. In this study, an initial “reasonable range” for each hyper-parameter in the FC + encoder–decoder modules was determined through trial and error, guided by previously published models,^26^^,^^27^ as listed in Table 3. The optimal hyper-parameter combination was then determined using fivefold cross-folding for internal validity. The average loss of the five folds was used as an overall metric of each model’s performance. The hyper-parameters used in the U-Net module were replicated from Ref. 27 in the interest of time.

**Table 3:** Hyper-parameter grid-search parameters for the DL-DOT model. Bold indicates the parameters of the best performing DL model, which was used for validation and evaluation.

FC layer activation	ReLu, **TanH**
Conv layer activation	**ReLu**, TanH
Number of 3D convolutional layers in encoder–decoder module	2, 3, **4**
Number of filters per conv layer	16, 32, **64**
Dropout rate	0, **0.2**, 0.4, 0.6, 0.8
Learning rate	0.1, 0.01, **0.001**, 0.0001, 0.00001

#### 2.4.5 Activation functions

In each layer of a neural network, a nonlinear activation function is applied to the layer’s output values, enabling deep networks to emulate complex nonlinear functions. In the original FDU-net, a rectified linear unit [ReLu, given by max(x,0)] activation was applied after every layer, including the initial FC layer, which, as explained in Sec. 2.4, acts as a generalized inverse operator. To estimate tissue absorption from CW intensity data, this choice makes sense as the effect of absorption on measured intensity is monotonic. The FC layer can therefore estimate voxel-wise perturbation from a baseline absorption via a weighted sum of the input values. However, in the case of multi-parameter reconstruction using both intensity and phase components of the FD measurement, the relationships between scattering and intensity, scattering and phase, and intensity and phase are known to be non-monotonic depending on the position of the voxel relative to the source and detector. It was therefore hypothesized that a function such as hyperbolic tangent (TanH) (given by $\frac{e^{x}-e^{-x}}{e^{x}+e^{-x}}$), which allows both positive and negative output values, would be more suitable than ReLu for conditioning the FC layer output. This prediction was born out in the grid-search results, which indicate that the most effective combination of activation functions was TanH for the FC layer and ReLu for the following convolutional layers, which is unique to this work for multi-parameter recovery.

#### 2.4.6 Training

The DL model was trained in a two-stage process. First, the FC + encoder–decoder modules were trained, and the weights and biases were frozen. The U-Net module was then trained separately on the CNN output and the ground truth volumes. Models were built and trained in Python using Keras DL API. All models were trained using the Adamax optimizer, and both stages of training were allowed to run up to 500 epochs, with an early-stopping mechanism that was activated if the validation error did not decrease at all over 50 epochs.

## 3 Evaluation

**Fig. 4:**
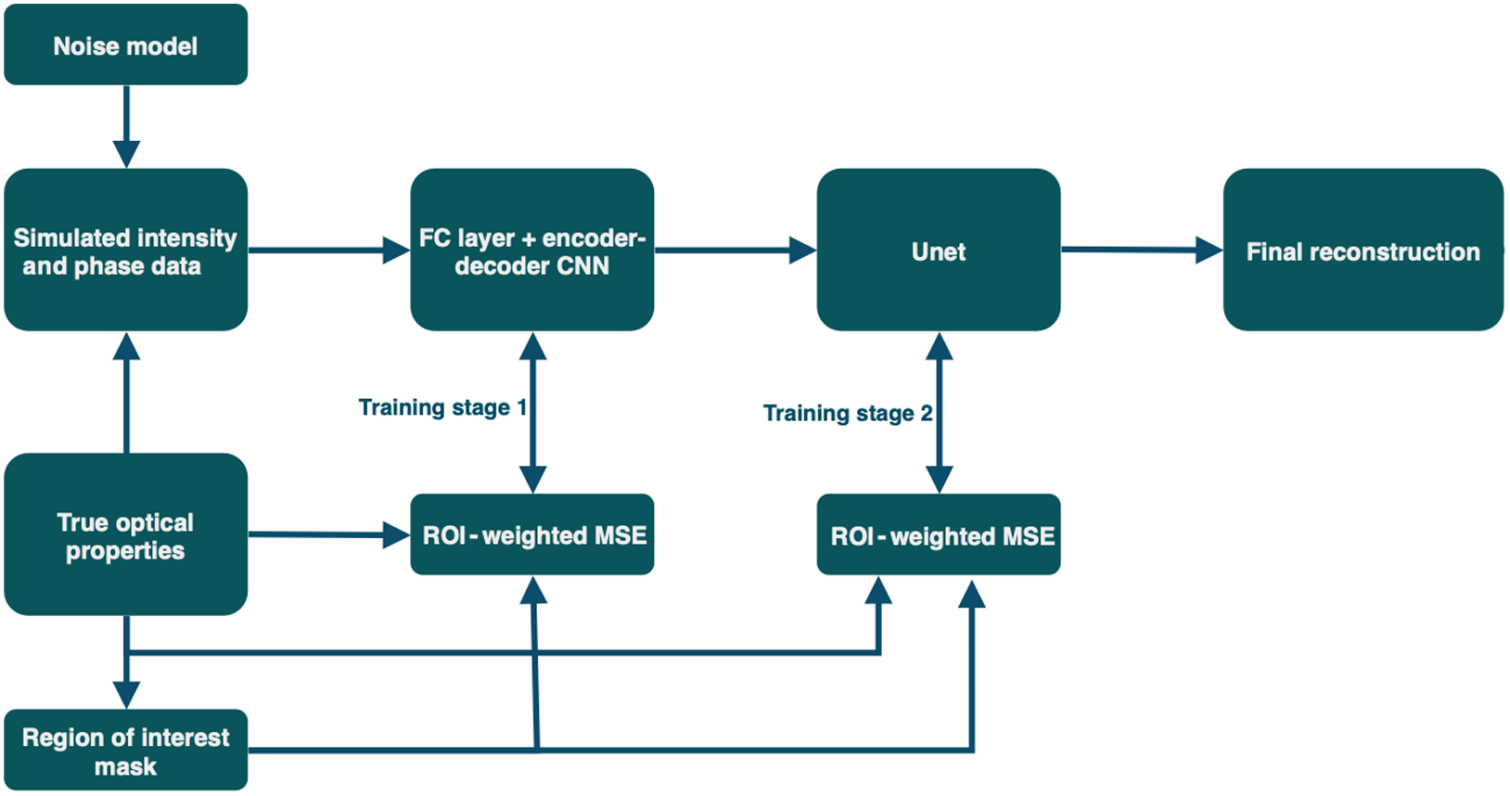
Diagram of the DL-DOT pipeline.

**Fig. 5:**
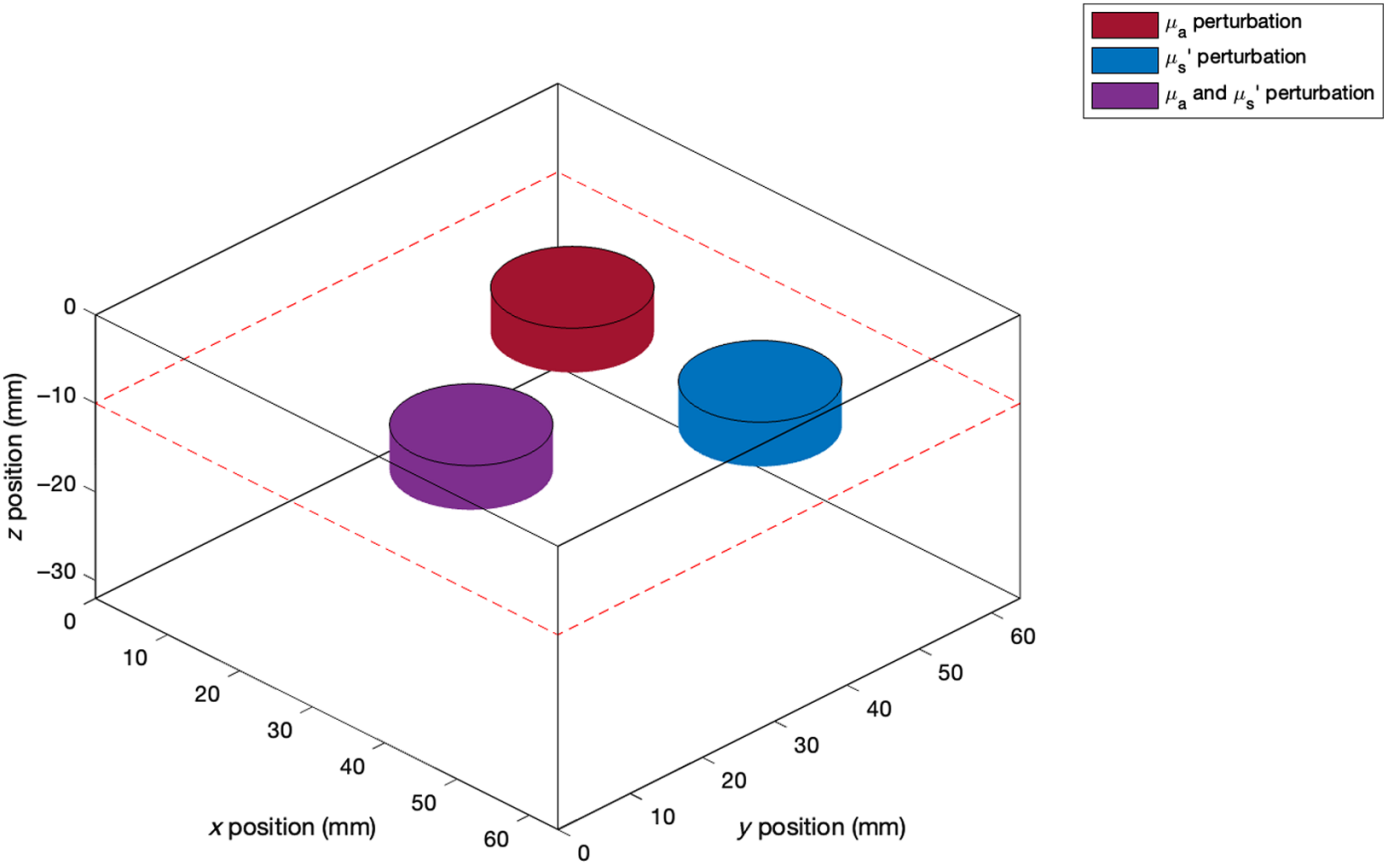
Schematic example of a manually designed test volume with three disk-shaped anomalies at a 10 mm depth.

A second simulated dataset of manually designed test volumes was used to demonstrate parameter separation and depth sensitivity of the proposed DL-DOT algorithm, compared with those of the model-based algorithm (FEM-DOT), keeping the size and shape of anomalies constant (Fig. 4). 150 test volumes were generated—30 for each of five discrete anomaly depths: 2.5, 5, 7.5, 10, and 15 mm. Three disk-shaped anomalies (radius = 8 mm, thickness = 5 mm) were added to each test volume: one in which only $\mu_{a}$ was perturbed, one in which only ${\mu_{s}}^{\prime }$ was perturbed, and a third in which both were perturbed, as illustrated in Fig. 5. The background optical properties and anomaly contrasts were sampled from the same distributions as the training data (Table 1), and realistic noise was added using the same system-derived model.^34^

The DL and FEM reconstructions of each test example were evaluated via four quantitative metrics: the MSE indicates the overall accuracy of the absolute optical property values; the Sørensen–Dice coefficient (SDC) indicates the spatial similarity of the reconstructed anomalies (closer to 1 is better) and is given by

SDC(y^anom,yanom)=2|y^anom⋂yanom||y^anom|+|yanom|, (4)

where y^anom is the anomaly mask of the reconstructed volume (calculated as voxels >0.5 in the min-max normalized volume) and $y_{\mathrm{anom}}$ is the ROI mask of the ground truth volume.

The contrast ratio (CR) represents the ratio between the reconstructed anomaly/background contrast and the ground truth (closer to 1 is better); it is given by

CR(y^,y,μ)=⟨y^μ(μ)⟩⟨y^bg(μ)⟩/⟨yμ(μ)⟩⟨ybg(μ)⟩, (5)

where μ denotes the optical property under consideration ($\mu_{a}$ or ${\mu_{s}}^{\prime }$); $y^{\left(\mu_{a}\right)}$ and y^(μ) are the corresponding channels of the ground truth and reconstructions volumes, respectively; the μ and bg subscripts indicate the perturbation and background voxels of those volumes, respectively; and CT indicates the CR of the opposite optical parameter in single parameter anomalies (closer to 0 is better), which is given by

(y^,y,μ)=|⟨y^μ−1(μ)⟩⟨yμ−1(μ)⟩−1|, (6)

where $\mu^{-1}$ indicates the optical parameter that is not under consideration.

### 3.1 Experimental Validation

**Fig. 6:**
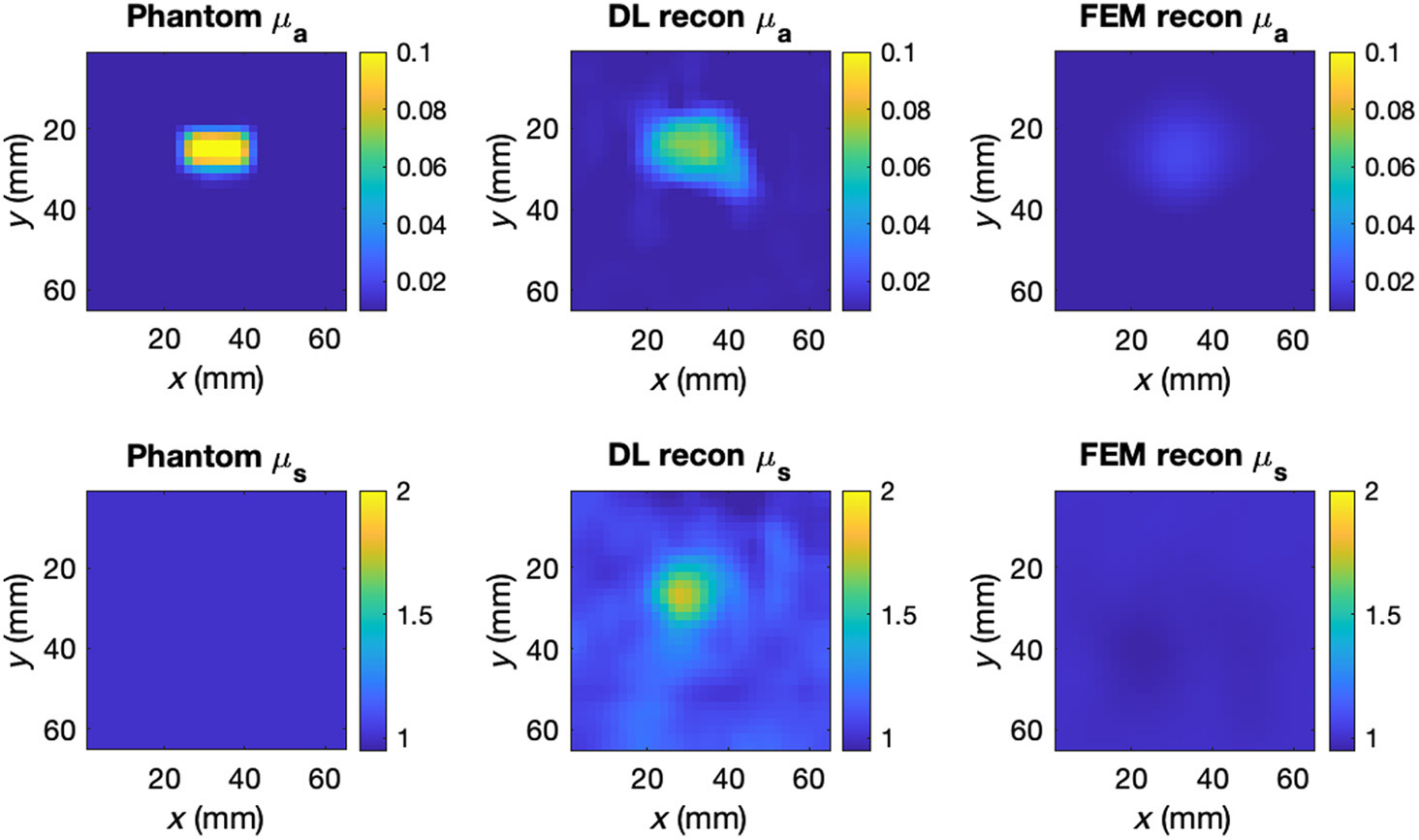
Cross-sections of the central scan region of phantom 1 and the corresponding DL and FEM reconstructions at 830 nm and 15 mm depths.

**Fig. 7:**
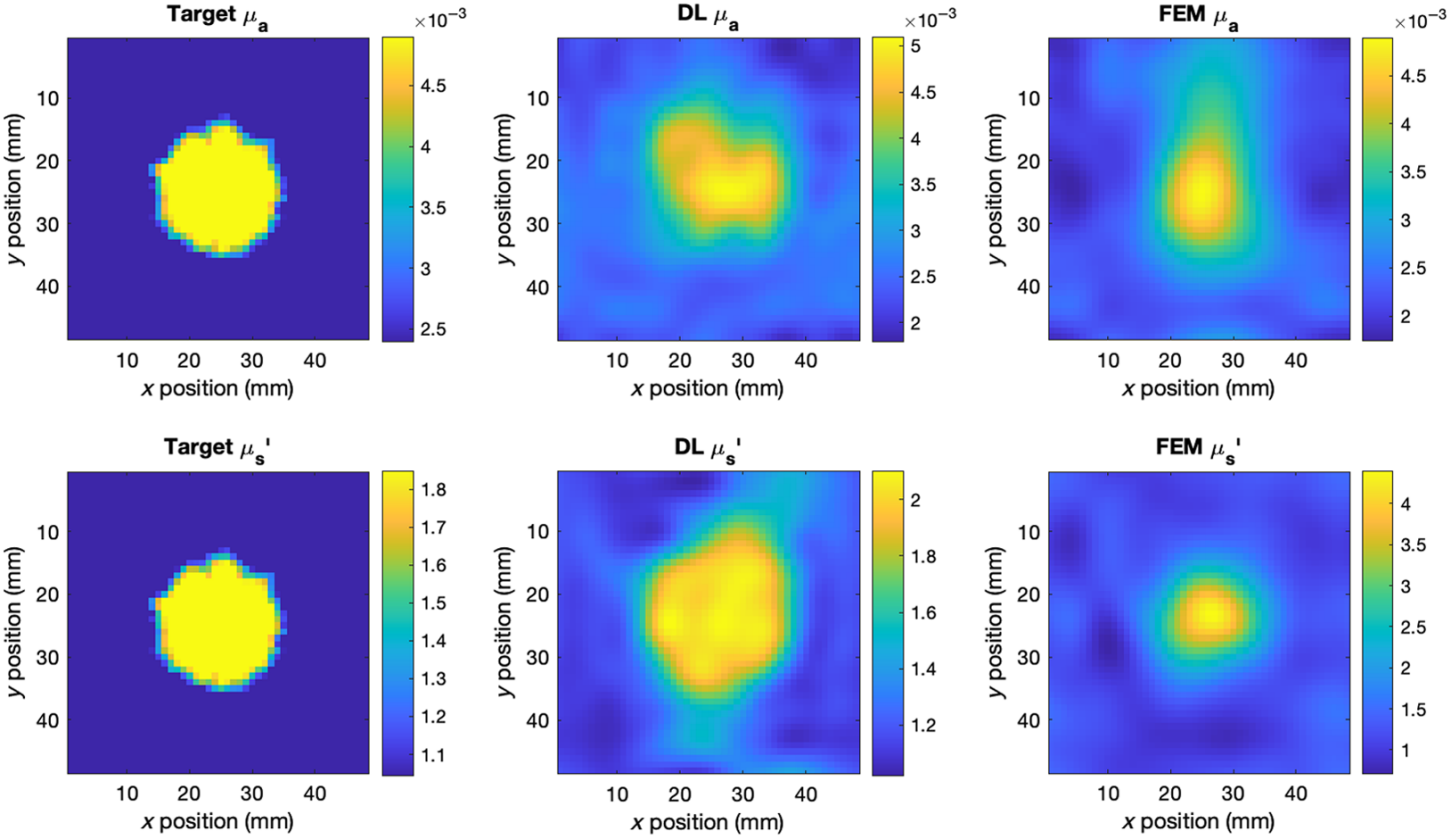
Cross-sections of the central scan region of phantom 2 and the corresponding DL and FEM reconstructions at 830 nm and 10 mm depths.

**Fig. 8:**
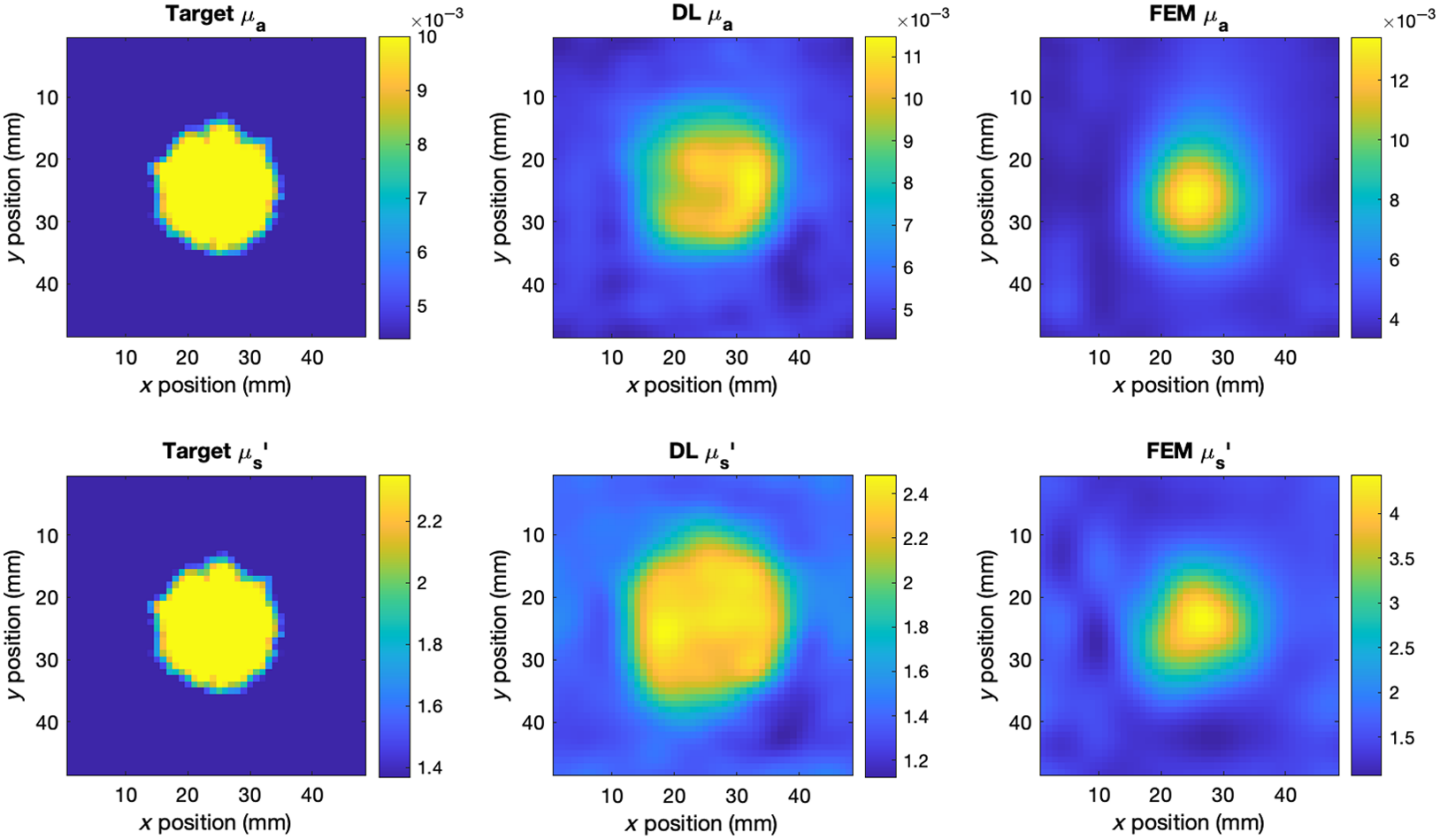
Cross-sections of the central scan region of phantom 1 and the corresponding DL and FEM reconstructions at 690 nm and 10 mm depths.

The two models were used to reconstruct the absolute optical properties of the physical phantoms described in Sec. 2.2, from the data shown in Fig. 3. Because the absorption contrast of the anomaly in phantom 1 was far greater than that represented in the biologically realistic synthetic data, a second training set was generated to train the DL model for phantom reconstruction, with the same randomization parameters except the range of $\mu_{a}$ perturbations, which was increased to 8 to 12 times the background value. The DL model was retrained following the same procedure, as described in Sec. 2.4.6. Cross-sections of the FEM and DL reconstructions of the phantoms are shown in Figs. 6–8. Only the optical properties corresponding to 830 nm measurements are shown for phantom 1 as the optical properties at 690 nm are not known.

## 4 Results

**Fig. 9:**
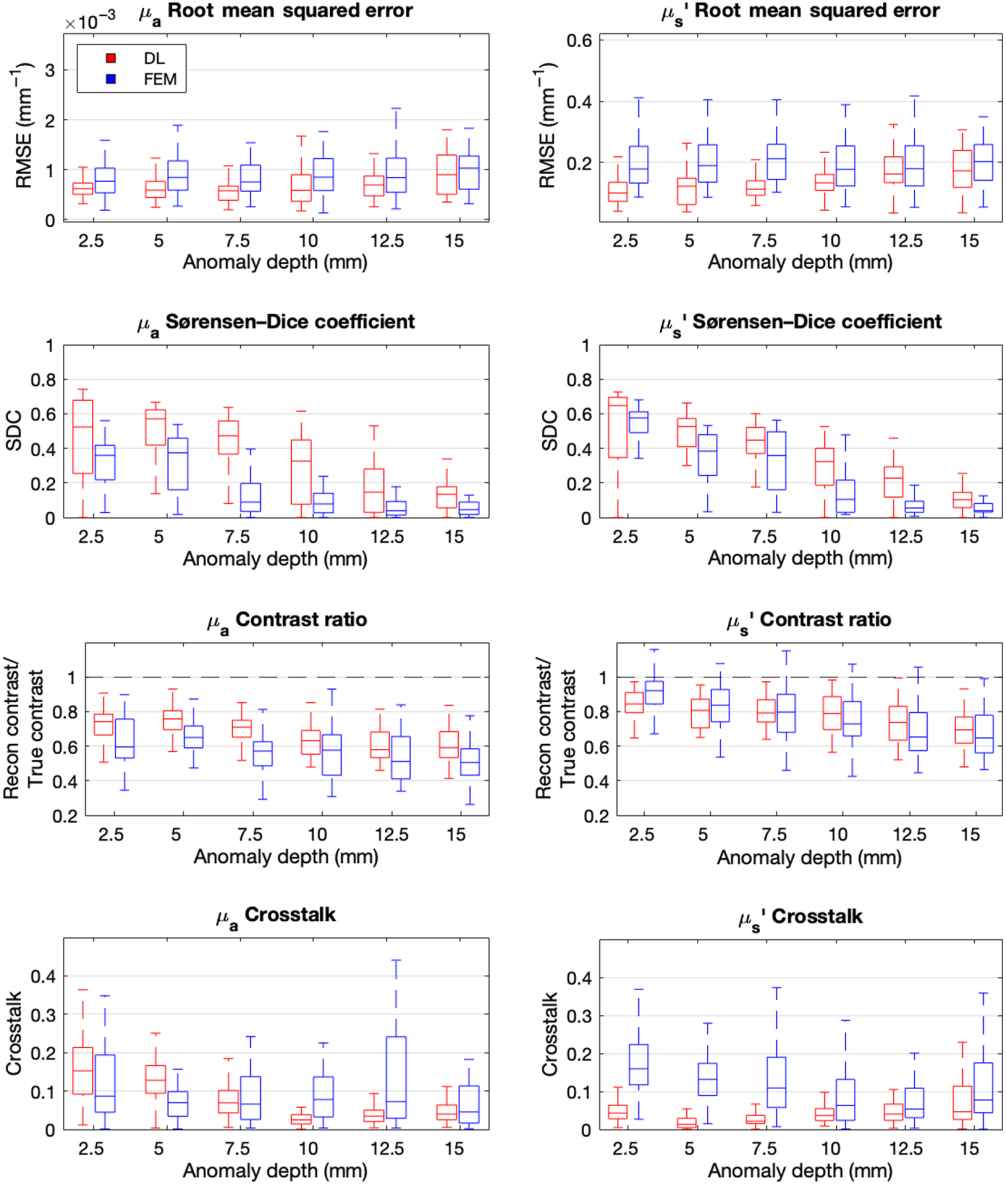
Average metric scores for normalized DL and FEM reconstructions grouped by anomaly depth.

Figure 9 shows the evaluation metrics calculated for the simulated test volumes for both models separated by inclusion depth.

A paired t-test was applied for each evaluation metric to test for statistical significance between the results for the two models across all anomaly depths. The DL model achieved a 12.4%±45.8% reduction in $\mu_{a}$ RMSE (p<0.0001) and a 23.1%±40% reduction in ${\mu_{s}}^{\prime }$ RMSE (p<0.0001), suggesting more precise voxel-by-voxel reconstructions. These results were significant at an alpha level of 0.05.

The observed differences in $\mu_{a}$ and ${\mu_{s}}^{\prime }$ SDC, $\mu_{a}$ CR, and $\mu_{a}$ and ${\mu_{s}}^{\prime }$ CT were also found to be significant (p<0.0001 in all cases) and in favor of the DL model. However, the difference in ${\mu_{s}}^{\prime }$ CR was not found to be significant (p=0.099).

The SDC was consistently higher in the DL reconstructions across all inclusion depths and both parameters.

The optical contrast of both parameters was generally underestimated by both models compared with the ground truth volumes. However, both $\mu_{a}$ and ${\mu_{s}}^{\prime }$ contrasts were higher and more consistent for the DL reconstruction, with the only exception being shallow (≤7.5  mm) ${\mu_{s}}^{\prime }$ inclusions.

The vast majority of both models’ reconstructions presented some degree of CT between the two optical parameters. Interestingly, the direction of CT in the DL reconstructions is modulated by anomaly depth; scattering perturbations were misattributed to absorption at lower depths; and vice versa at greater depths. By contrast, for the FEM model, the two are not clearly related.

The reconstruction metrics calculated with respect to the physical phantom reconstructions are shown in Tables 4–6.

**Table 4:** Evaluation metrics for DL and FEM reconstructions of phantom 1 at 830 nm.

	DL model		FEM model	
	$\mu_{a}$	${\mu_{s}}^{\prime }$	$\mu_{a}$	${\mu_{s}}^{\prime }$
**RMSE** (${\mathrm{mm}}^{-1}$)	0.01115	0.48176	0.01156	0.08305
**SDC**	0.39705	—	0.06577	—
**CR**	0.79577	—	0.16571	—
**CT**	—	0.970537	—	0.12838

**Table 5:** Evaluation metrics for DL and FEM reconstructions of phantom 2 at 830 nm.

	DL model		FEM model	
	$\mu_{a}$	$\mu_{s}\prime$	$\mu_{a}$	$\mu_{s}\prime$
**RMSE** (${\mathrm{mm}}^{-1}$)	0.0006	0.2703	0.00119	0.32107
**SDC**	0.62978	0.6423	0.10665	0.36969
**CR**	1.0934	1.0812	1.3229	2.2697
**CT**	—	—	—	—

**Table 6:** Evaluation metrics for DL and FEM reconstructions of phantom 2 at 690 nm.

	DL model		FEM model	
	$\mu_{a}$	$\mu_{s}\prime$	$\mu_{a}$	$\mu_{s}\prime$
**RMSE** (${\mathrm{mm}}^{-1}$)	0.00137	0.26593	0.00152	0.31914
**SDC**	0.60832	0.64658	0.40088	0.46681
**CR**	1.0451	1.0885	1.223	1.8557
**CT**	—	—	—	—

### 4.1 Time Efficiency

The computation time of the two models was measured on a Linux system with an Intel(R) Xeon(R) Bronze 3106 CPU processor (1.70 GHz), 64 GB of RAM, and an onboard GPU (Tesla V100-PCIE) with 16GB HBM2 memory and 5120 CUDA cores.

The time taken for a single FEM reconstruction was 228±100  s (the average number of iterations before convergence was 5.6±3.6). The DL training dataset took a total of 41 h and 26 min to generate all 11,000 examples. Training time for the DL model was 1 h and 37 min (37 min for the FDnet and 1 h for the U-Net). The time taken for a single DL reconstruction was 0.0165±0.0018  s.

### 4.2 Computational Order Analysis

The FEM’s computational order is primarily influenced by the number of free variables (nodes) in the mesh, and the number of iterations per reconstruction, suggesting an approximate computational complexity of O(n·i), where n is the number of mesh nodes and i is the average iterations to convergence. By contrast, the DL model’s computational complexity is determined by the network architecture and can be estimated as O(1).

## 5 Discussion

### 5.1 Interpretation of Results

In this work, a deep CNN was used to reconstruct absolute 3D $\mu_{a}$ and ${\mu_{s}}^{\prime }$ values of physical and digital phantom tissue models directly from the intensity and phase components of frequency-domain NIR measurements. Although previous works have harnessed DL to solve the DOT inverse problem, both for the 2D multi-parameter case^33^ and the 3D absorption-only case,^27^ this is the first demonstration of DL-DOT for the 3D multi-parameter case. The benefits of 3D DOT and reconstructing scattering in addition to absorption, as discussed in Sec. 1, are substantial in the context of breast tumor characterization, and although multi-parameter reconstruction incurs additional obstacles, including the possibility of CT, it is a promising alternative to the CW paradigm, which requires the false assumption of constant scattering.

The evaluation metrics for the two test sets (simulated and experimental) were generally coherent, supporting the use of simulated data to evaluate the algorithms’ performance. Furthermore, the average recovered background $\mu_{a}$ and ${\mu_{s}}^{\prime }$ values of the physical phantoms were within 10% of the ground truth, indicating that the calibration process effectively transforms the data between system and simulation measurement domains.

Overall, the results indicate the same benefits of the DL technique observed elsewhere with absorption-only models.^26^^,^^27^ The DL reconstructions generally had lower RMSE, higher spatial similarity (as measured by SDC), and more accurate optical contrast compared with conventional FEM-DOT using the same test data. Furthermore, analysis of the reconstructions of the simulated volumes revealed that the performance of the DL model was more stable, as evidenced by the reduced variance in the metric outcomes, and that the falloff in performance as a function of anomaly depth was smoother and more reflective of the expected sensitivity falloff of $\mu_{a}$ and ${\mu_{s}}^{\prime }$, indicating that its performance is limited by the underlying physics of the problem rather than the model itself. In the reconstructions of phantom 2, which was designed for this study to emulate realistic optical properties of human breast tissue (see Table 2), the DL reconstruction was better than the FEM across all metrics for both optical properties and at both wavelengths.

The primary contribution of this study was to evaluate the effectiveness of the DL-DOT algorithm for reconstructing absolute reduced scattering in addition to absorption. It was observed that the difference in normalized RMSE distributions between the two models was significantly greater in the ${\mu_{s}}^{\prime }$ reconstruction than the $\mu_{a}$, suggesting an additional benefit of DL for multi-parameter DOT beyond that observed in previous works; this can be attributed to the inherent difficulties of reconstructing absolute scattering values using an iterative algorithm. First, because scattering has a diffusive effect on the sensitivity distributions of both $\mu_{a}$ and ${\mu_{s}}^{\prime }$, varying scattering adds additional nonlinearity to the inverse problem compared with the constant scattering case and a greater dependence on higher-order derivatives, which are often ignored in the FEM approach to linearize the update calculation. Second, the lower sensitivity of both intensity and phase with respect to biologically relevant scattering and the sharper falloff in sensitivity as a function of depth both contribute to a significantly lower SNR in the FD measurements with respect to scattering perturbations. The DL model has improved robustness to noise as the convolutional layers automatically learn the specific distribution of noise in the training data compared with the FEM model, which relies on manually chosen matrix regularization and a smoothing filter to avoid over-fitting, both of which incur a direct cost in terms of localization and contrast. However, shallow perturbations in ${\mu_{s}}^{\prime }$ were identified as a specific cause of error for the DL model, evidenced by both the lower contrast in ${\mu_{s}}^{\prime }$ and the higher CT in $\mu_{a}$ compared with the corresponding FEM reconstructions (Fig. 9). This may be due to the inherent uncertainty regarding the position of the anomaly introduced by scattering close to the source/detector and the differing effects of this uncertainty on the convergence of the two algorithms. The DL model uses a single set of parameters to minimize voxel-wise error across all training examples and therefore effectively predicts a weighted average of possible solutions for a given input—positional uncertainty therefore produces a diffusive effect in the reconstruction. In the FEM algorithm, however, the error is calculated between two sets of measurements, and the model weights (the Jacobian) are updated at each iteration given the current optical property estimate—the algorithm therefore converges in a “depth-first” manner toward a single most likely solution and is less effected by the uncertainty introduced by scattering. Knowing this specific weakness, it may be possible to adapt the DL model to improve its performance in cases in which anomaly localization is uncertain, for example, by tailoring the ${\mathrm{MSE}}_{\mathrm{ROI}}$ loss function to penalize false-positive anomalies in the shallow region of the reconstruction, thereby increasing sensitivity to subtle indications of anomaly position and contrast.

Parameter separation in DOT is vital in the context of breast imaging, both to improve the accuracy of recovered $\mu_{a}$ and therefore of the subsequently resolved chromophore concentrations and to provide an indication of changes in tissue substructure through the scattering parameter. In all of the test cases in which the optical properties were within a biologically realistic range for breast tissue (phantom 2 and simulated volumes), the results indicate that the DL model can more effectively separate the effects of absorption and scattering compared with the FEM reconstruction, which was more impacted by CT between the parameters. However, in the case of phantom 1, the DL reconstruction exhibited a significantly greater degree of CT and a corresponding higher ${\mu_{s}}^{\prime }$ RMSE compared with the FEM reconstruction. This may be due to the relatively greater background absorption and anomaly depth in phantom 1 compared with the other test volumes: during the development of the multi-parameter DL model, it was noted that “hallucinated” anomalies sometimes occur in the reconstructions when signal contrast is low and that this tendency is greater when the average number of anomalies in the training data is greater. There may therefore be a benefit of the model-based approach specifically in cases in which only low SNR measurements are available. It should also be noted that there may be genuine scattering changes in phantom 1 that were unaccounted for in the ground-truth target; this is caused by imperfect contact between the phantom and the movable anomaly rod. In this work, the only available phantom with biologically realistic optical properties (phantom 2) contained a single anomaly characterized by a perturbation in both $\mu_{a}$ and ${\mu_{s}}^{\prime }$ and therefore did not give a clear indication of parameter separation. In future work, it would be beneficial to test models on physical phantoms in which $\mu_{a}$ and ${\mu_{s}}^{\prime }$ are separately perturbed within a biologically relevant range, to separate the effects of the model choice, optical properties, and measurement paradigm (simulated or experimental) on parameter separation.

Both models consistently underestimated the contrast of the simulated anomalies. Low contrast is a common issue for model-based reconstruction due to the inherent trade-offs and has therefore been explicitly addressed in the DL model design, using a region-of-interest weighted loss function during training. The DL model recovered more accurate contrast for all test cases, except for simulated examples with shallow (2.5 to 7.5 mm) ${\mu_{s}}^{\prime }$ perturbations.

### 5.2 Neural Network Design

This work introduced three specific developments to the FDU-net as described in Ref. 27 to improve the performance for multi-parameter recovery using FD data. First is the use of z-score rather than min-max normalization to preprocess the FD measurements—this was found to reduce overall RMSE in the validation data by 13% and the average number of epochs taken for convergence (although the latter was not systematically measured). It is noted that this choice was made at the validation stage, at which only RMSE was evaluated, and it is possible that using min-max or another normalization method may benefit other metric scores such as anomaly contrast. Second is the use of the TanH function as opposed to ReLU for the FC layer—to account for positive and negative sensitivity in the FD measurements with respect to absorption and phase—which reduced ${\mathrm{MSE}}_{\mathrm{ROI}}$ loss by 10% in a direct comparison using fivefold cross-validation. Third is the combination of the $\mu_{a}$ and ${\mu_{s}}^{\prime }$’ ROI masks that penalize CT artifacts, which reduced ${\mathrm{MSE}}_{\mathrm{ROI}}$ by 25% in examples with single parameter inclusions.

Notwithstanding these improvements, the adapted FDU-net presented in this work represents a canonical DL architecture for an inverse imaging problem: an initial FC layer used as a mapping function from measurement space to voxel space, followed by a structure of convolutional layers for spatial feature extraction, followed in this case by a U-Net module to integrate high- and low-level features to produce cleaner DOT images. This style of architecture has the benefit of being arbitrarily adaptable to different measurement protocols and tissue geometries with minimal implementation changes. However, because the FC layer learns a weight for each available measurement, voxel, and optical parameter independently, any single trained network is not generalizable to different probe configurations or scan protocols, potentially leading to a significant time cost for case-by-case data generation and network training. This “probe configuration dependency” is unlikely to be overcome with the current DL-DOT paradigm but may be with a different style of architecture such as a vision transformer (ViT) or graph neural network, which allows for ad hoc encoding of arbitrary measurement sequences and node positions. It is possible however that an adapted FDU-net architecture could improve the performance or efficiency of a particular geometry or scan protocol. For example, the reflectance geometry and moving probe used in this work ensure structural similarity between the sensitivity distributions of all measurements of the same source/detector separation, inevitably leading to redundancy in the FC layer. Implementing parameter-sharing techniques (e.g., 2D convolution over spatially resolved measurements) to enforce a generalized spatial mapping from measurement to voxel space could reduce redundancy in the network and enable more efficient training and a significant reduction in the number of parameters. This is highly desirable in the context of handheld NIR devices, for which processing power and memory space are limited by the controlling device, often a tablet or smartphone. The trade-offs in network size, training efficiency, and inference time in the context of handheld DOT will be the subject of future research.

### 5.3 Toward Real-Time Clinical Imaging with DOT

The DL and FEM models described in this work represent two qualitatively different approaches to DOT, each with distinct benefits and drawbacks. The FEM approach relies on an explicit physics-based forward model and can therefore be utilized with no priors regarding the characteristics of the target volume besides a known geometry and an initial estimate of optical properties. However, because the iterative optimization algorithm linearizes a highly nonlinear problem, it is likely to converge in a local minimum, leading to errors in the reconstruction. By contrast, the DL approach can effectively model the complexity of the inverse problem for a given geometry, but it has no explicit representation of the physical system that it intends to invert. It relies entirely on statistically extracted features based on specific training data used, meaning that prior distributions of volume properties, such as ranges of optical properties, number, size, and shape of anomalies, encoded in the training data are automatically learned by the model and bias its reconstructions. This is both a strength and a weakness of the DL method: the bias reduces the number of possible solutions but also means that the model may fail to reconstruct examples with properties that are not represented in the training dataset (this is why, for example, an entirely new training set was required to reconstruct phantom 1, the optical properties of which lay outside the range simulated in the original training set). Generalizability is a common limitation of DL models across domains and can lead to unpredictable errors even in extremely advanced DL models. Such failure modes are clearly unacceptable in the context of disease monitoring or diagnosis, so although DL-DOT may provide a valuable clinical guide, it is unlikely to be reliable enough to inform critical decision-making without significant progress in interpretability or used in combination with a physics-based model (as recently demonstrated^41^).

From a clinical point of view, it can be argued that the most notable distinction between the DL-DOT and FEM-DOT methods is the high reconstruction speed and automatability of DL. This is increasingly salient considering the recent advances in FD-DOT hardware, as discussed in Sec. 1, especially the trend toward lightweight, handheld devices that can be run from a tablet or smartphone^13^ and the fact that one of the key advantages of DOT imaging compared with other modalities is its relative speed and usability. As has been reported frequently elsewhere, the time and computational cost of DL is incurred primarily in the data generation and training phases, enabling significantly faster reconstruction. In this study, the DL method reduced the inference time by approximately four orders of magnitude compared with the FEM method (0.02 s versus 3.8 min). Deng et al.^27^ found slightly different times (0.02 s versus 12 min)—possibly due to differences in the input and output data size, mesh resolution, or GPU speed—but a similar reduction of four orders of magnitude. In addition, the inference time of a trained DL model is constant, whereas the iterative FEM optimization depends on the number of iterations before the stopping criteria are met. Even without further optimization, assuming a 10 Hz refresh rate brings DOT into the same range of temporal resolution as some modern ultrasound systems. Furthermore, the FEM method relies on manually chosen parameters such as the regularization technique, number of iterations, and design of the smoothing filter. In this study, the FEM parameters were chosen to maximize performance over a labeled validation dataset and then kept the same for all test reconstructions. Although the parameters could certainly be tuned individually for each example based on experience, expert intuition, or prior information, to produce a higher quality reconstruction, this is impractical in a clinical setting, where prior information is rarely available and the medical professional using the system is unlikely to be an expert in the relevant computational techniques. By contrast, DL-DOT front-loads algorithmic choices in the data generation and training stages, meaning that once the model is trained, reconstruction can be fully automated at the point of use. Furthermore, the same DL model can be used to recover absolute $\mu_{a}$ and ${\mu_{s}}^{\prime }$ for multiple wavelengths (as demonstrated with phantom 2), thereby enabling real-time estimation of both absolute chromophore concentrations and scattering properties (particle size and density)^42^ with a single scan. Considered together, these advantages suggest a potential new use-case for FD-DOT: high-speed 3D imaging, which could provide an immediate visualization following a scan, or even real-time feedback for a clinician administering a scan with a handheld device. It is noted that the same measurements used for real-time DL-DOT can be stored and subsequently used for FEM or hybrid reconstruction, conferring the benefits of both techniques from a single set of measurements.

### 5.4 Limitations

First, and as previously noted, although simultaneous dual-wavelength $\mu_{a}$ and ${\mu_{s}}^{\prime }$ reconstruction has been demonstrated, recovery of pathologically relevant chromophores (e.g., oxygenated and deoxygenated hemoglobin) has not. It is difficult to quantify the benefit of increasing optical property accuracy without performing the conversion to chromophore values, and this should be explored in subsequent work.

Second, the simplified tissue models (cuboid slabs with homogeneous backgrounds and uniformly perturbed anomalies) used to train and evaluate the models in this study do not capture the full range of tissue characteristics expected in real human tissue. Rather, these models were designed to isolate the effects of optical property contrast and anomaly depth. Although some studies have indicated that DL-DOT models can generalize beyond the specific characteristics of the training data, this is an exception to the general rule; typically, DL models excel in specialized imaging tasks but are prone to errors when confronted with “out of distribution” examples. In the case of DOT, spatial priors such as optical property distributions and anomaly shapes and sizes are encoded in the training data and automatically learned and reproduced by the model. Consequently, the trained DL model evaluated in Sec. 3 is not expected to generalize to more complex anomaly shapes than those present in the training dataset. However, it is likely that an identical architecture could recover more complex anomaly shapes and smooth yet non-uniform background volumes such as breast tissue, given an appropriately designed training dataset. Developing effective datasets for data-driven medical imaging is challenging compared with traditional DL applications such as image classification or language translation due to the unavailability of ground-truth training examples. Nevertheless, techniques demonstrated elsewhere, such as incorporating structural information from X-ray mammography to produce more realistic simulated examples, may offer benefits over the simplified volumes used in this study.

No work has yet addressed the related question of the generalizability of DL-DOT models with respect to individual and population-level physiological differences. In this work, the optical properties in the simulated DOT dataset were sampled from a continuous distribution. In reality, factors such as age and ethnicity affect tissue density and skin pigmentation, producing distinct differences in optical property distributions among demographic groups. Accounting for these differences by narrowing the biological priors in the training data may provide more accurate reconstructions by reducing the range of possible solutions, but it introduces additional methodological considerations such as defining appropriate groups and classifying patients within them. At the extreme end of this spectrum, unique datasets could be generated for individual patients by co-registering 3D scans or with an atlas model, which may be especially beneficial for patients with unusual requirements in terms of scan location or body shape, that are unlikely to be accurately reconstructed by a model trained on a population-level dataset.

## 6 Conclusion

This work evidenced DL-DOT for simultaneous recovery of 3D absorption and scattering coefficients. In addition to preserving the previously demonstrated benefits of DL-DOT, including dramatically reduced reconstruction time and more accurate absorption recovery, the proposed DL model—trained exclusively on simulated FD-NIR data—infers specific advantages for multi-parameter reconstruction, including improved parameter separation and accurate recovery of tissue scattering, which poses a fundamental obstacle to the classic FEM-based approach. The results support the potential of DL for enhancing the efficiency and accuracy of FD-DOT imaging and the possibility of harnessing DOT for real-time handheld breast imaging in a clinical setting.

## Data Availability

The data presented in this article are publicly available in “multiparameter_DOT_dataset” at https://doi.org/10.5281/zenodo.10379351. The code implementation of the model described in this work may be made available upon reasonable request.
